# Impact of molar incisor hypomineralization on oral health-related quality of life, dental fear and anxiety in Swedish children

**DOI:** 10.2340/aos.v84.43856

**Published:** 2025-06-11

**Authors:** Adnan Hajdarević, Birgitta Jälevik, Emina Čirgić, Agneta Robertson, Nina Sabel

**Affiliations:** a Department of Pediatric Dentistry, Institute of Odontology, Sahlgrenska Academy, University of Gothenburg, Gothenburg, Sweden; b Folktandvården Björkekärr, Public Dental Service, Region Västra Götaland, Gothenburg, Sweden; c Institute of Odontology, Sahlgrenska Academy, University of Gothenburg, Gothenburg, Sweden; d Department of Orthodontics, Folktandvården Stockholms län AB, Folktandvården Eastmaninstitutet, Stockholm, Sweden; e Clinic of Pediatric Dentistry, Public Dental Service, Region Västra Götaland, Gothenburg, Sweden

**Keywords:** Developmental defects of enamel, dental enamel hypomineralization, pediatric dentistry, Index

## Abstract

**Objectives:**

The aims this research were to analyze self-reported oral health-related quality of life (OHRQoL) and dental fear and anxiety (DFA) in 11-year-old patients after either restorative treatment or after extraction of first permanent molars (FPM) affected by severe molar incisor hypomineralization (MIH). The research question focused on whether these treatments lead to different outcomes of DFA and OHRQoL over time.

**Materials and methods:**

GuREx-MIH, a multicenter trial, was conducted involving 83 children aged 6–9 years who were diagnosed with severe MIH in FPMs. Patients were randomly assigned to receive either restorative treatment with resin composite or extraction. Patient comfort was assessed through OHRQoL and DFA, using the Swedish version of the Child Perceptions Questionnaire (CPQ_11-14_) and the Children’s Fear Survey Schedule-Dental Subscale (CFSS-DS), which were administered before treatment (T0) and at follow-up when patients were 11 years old (T1). Descriptive statistical analyses were conducted and comparisons between the restorative and extraction groups were performed using T-tests.

**Results:**

A total of 79 patients completed the study, with 43 allocated to restorative treatment and 36 to extraction. At follow-up, the mean OHRQoL score was 8.9 (standard deviation [SD] 7.3) for patients in the restorative group and 9.6 (SD 6.7) for those in the extraction group (*p*: 0.337, *T*-test). The mean DFA score was 21.5 (SD 5.5) for the restorative group and 23.1 (SD 6.8) for the extraction group (*p*: 0.130, *T*-test).

**Conclusions:**

Restorative treatment and extraction of FPMs affected by MIH lead to similar impact on DFA and OHRQoL at 11 years of age.

## Introduction

Molar incisor hypomineralization (MIH) is a condition characterized by enamel defects of unknown etiology despite extensive research over the past two decades. As per definition, MIH affects one to four first permanent molars (FPM) and often involves the permanent incisors. Clinically, affected teeth with demarcated opacities and in severe cases, post-eruptive breakdown may occur [1]. Globally, MIH affects approximately 14% of children [2], with post-eruptive breakdown occurring in 21% of teeth with demarcated opacities [3]. Molars affected with MIH frequently cause hypersensitivity to cold food and drinks, air, and general dental discomfort [4]. In addition, there is an increased risk of caries development in MIH-affected teeth, particularly in those exhibiting post-eruptive breakdown [5]. Severe MIH often require intervention shortly after eruption and treatment needs are variable and depend on the severity of the defect and associated symptoms [6].

Despite the high prevalence of MIH, there remains a lack of consensus among dental professionals regarding the most appropriate treatment strategies, including decisions about extraction versus restauration and, in addition, the selection of restorative materials. Consequently, general dentists often seek the expertise of pediatric dentistry specialists prior to finalizing treatment plans. Common treatment approaches include composite restorations or extraction [7, 8].

The decision to restore or extract affected FPMs is influenced by multiple factors, including patient preferences, the extent of the condition, and long-term dental prognosis. While restoration can preserve the tooth, it may lead to repeated interventions over time, eventually requiring extraction. In contrast, extraction eliminates the need for future restorative treatments but may necessitate orthodontic management, especially in young patients where space closure is a consideration. The choice between these options should be guided by patient, mouth, and tooth-level factors, as well as input from both patients and parents [9].

Long-term studies indicate that the quality of restorative fillings in MIH-affected teeth is often inadequate, with a median lifespan of approximately 5 years [10]. Common challenges in restorative treatment include difficulties with achieving adequate anesthesia, which can lead to painful experiences during the treatment [11]. These complications, along with the frequent need for retreatment, may contribute to increased dental fear and anxiety (DFA) among patients with severe MIH compared to those without [12].

The prevalence of DFA varies from 13.3% to 29.3% depending on age group, gender, and sociodemographic aspects [13]. Dental care experiences are a common factor contributing to DFA across all ages [14]. The etiology of DFA is multifactorial, extending beyond dental care and influenced by personal and external factors. DFA is defined as an emotional fear response to potential threats during dental situations [15]. These experiences encompass both physiological and psychological aspects such as painful treatments, insufficient anesthesia, or the dental care environment, for example, sounds, smells, lighting, which can foster fear of dental visits [16]. In addition, the lack of control over what occurs during dental care plays a role [16].

A review by Jälevik et al. [17] found that patients with MIH experience significantly reduced oral health-related quality of life (OHRQoL), particularly concerning oral symptoms and functional limitations, compared to those without MIH. However, the study relied on generic OHRQoL measures rather than an MIH-specific instrument, which may limit the precision of the findings. OHRQoL considers a patient’s subjective assessment of their oral health and overall well-being, influenced by factors such as discolored anterior teeth, caries, and malocclusions [18 20].

The optimal treatment approach for severe MIH remains a subject of ongoing debate within the dental community, with limited evidence on the long-term impacts of different outcomes of various interventions, particularly from the patients’ perspective. Therefore, there is a critical need for prospective clinical studies to investigate whether restoration or extraction is the most appropriate course of action for teeth diagnosed with severe MIH. Furthermore, there is a gap in knowledge regarding how these treatments affect children’s quality of life and psychological well-being over time. Patient Reported Outcome Measures (PROMs), which evaluate both physiological and psychological aspects of patient experiences, are essential for assessing the effectiveness of treatment. This holistic approach provides a comprehensive understanding of treatment outcomes integrating clinical assessments with patient perspectives. The objectives of this trial were to evaluate self-reported OHRQoL and DFA among patients who underwent either restorative treatment or extraction of their FPM due to severe MIH.

## Material and methods

### Trial design

A multicenter trial was conducted to follow patients with severe MIH diagnosed in their FPM before treatment (T0) at 11 years of age (T1) and 15 years of age (T2). The GuREx-MIH project (**G**othenburg **U**niversity **R**estoration or **Ex**traction of First Permanent Molars due to Severe **MIH**) aimed to evaluate the outcomes of restoration and extraction treatment for severe MIH. This trial and the protocol were registered in ClinicalTrials.gov (registration number: NCT06228989).

Age-appropriate information was provided to all participating patients and their caregivers. Informed consent was obtained from the guardians after they were provided with comprehensive verbal and written information regarding the study. The study adhered to ethical standards as outlined by the World Medical Association Declaration of Helsinki and received approval from the Institutional Review Board (IRB) and the Swedish Ethical Regional Board in Gothenburg, Sweden (Dnr: 352-15).

### Patients

Patients were recruited from the Clinics for Pediatric Dentistry at the Public Dental Service in Region Västra Götaland, Region Östergötland, and the Department of Pedodontics at Malmö University. Referrals to the clinics were performed by general dentists for patients with MIH-affected FPMs that required treatment. Inclusion criterion was patients aged 6–9 years who presented at least one FPM diagnosed with MIH degrees 4 or 5 (Table 1). Exclusion criteria included the presence of dental agenesis, chronic systemic diseases, or functional impairments.

**Table 1:** Categorization of the first permanent molars with MIH by Hajdarević et al. [7], including patients’ symptoms.

xxx	Degree	Description	Meet the inclusion criteria
INTACT TOOTH	0	Sound enamel, or hypomineralization < 1 millimeter in diameter	no
MILD MIH	1	Demarcated opacities, without enamel breakdown, without symptoms	no
	2	Demarcated opacities, without enamel breakdown, with symptoms	no
MODERATE MIH	3	Hypomineralized enamel with enamel breakdown or atypical restauration ≤ 2 surfaces, without symptoms	no
	4	Hypomineralized enamel with enamel breakdown or atypical restauration ≤ 2 surfaces, with symptoms	yes
SEVERE MIH	5	Hypomineralized enamel with enamel breakdown or atypical restoration, > 2 surfaces	yes
	6	Hypomineralized enamel with enamel breakdown or atypical restoration, > 2 surfaces and/or extensive lesion (> ⅔ of the depth of the dentin)	no

### Sample size

The sample size was based on an alpha significance level of 0.05 and a power of 80%, aiming to detect a difference of 4 units (standard deviation [SD] 8.4) in the score of CPQ_11-14_ between patients allocated to restorative and extraction treatment groups, based on data from Jokovic et al. [21]. The sample size analysis indicated 70 patients in each treatment group.

### Randomization

Randomization was conducted by author B.J. using a computer-based random number generator in the mobile application The Random Number Generator [22]. Each patient was assigned a number between zero and nine, with even numbers allocated to restorative treatment and odd numbers to extraction. Allocation concealment was ensured by employing a computerized randomization system. The randomization sequence was generated independently to minimize selection bias.

Blinding was not possible in this study due to the nature of the interventions, as both restorative treatment and extraction were visibly distinguishable by both clinicians and patients. Consequently, it was not possible to blind either the participants or the treatment providers.

### Interventions

#### Baseline

At baseline (T0), dentists from the Clinics for Pediatric Dentistry underwent training and calibration for an accurate MIH degree using a photo assessment manual. Comprehensive oral examinations, including panoramic and lateral cephalometric radiographs, were conducted. Caries status was registered as the number of decayed, extracted, and filled teeth in the primary dentition (deft) [23]. Patients completed surveys on OHRQoL and DFA. Patients were randomly assigned to restorative treatment with resin composite or extraction. These treatments were conducted based on the randomization outcomes at the Clinics of Pediatric Dentistry. Sedation (benzodiazepine and/or nitrous oxide) or general anesthesia (GA) was administered when considered necessary, determined by the attending pediatric dentist.

#### Follow-up

At the 11-year follow-up (T1) overseen by author A.H., patients underwent oral examinations and the presence of MIH-related opacities on incisors was recorded. Panoramic radiographs were also taken. Patients completed questionnaires on OHRQoL and DFA. The 15-year follow-up (T2) is pending with future assessments planned.

#### Outcomes

OHRQoL was assessed using the Swedish version of the validated short-form CPQ_11-14_ [21]. Higher CPQ_11-14_ scores indicate a lower OHRQoL. The 16 questions of the survey represent four domains: Oral symptoms, Functional limitation, Emotional well-being, and Social well-being. Each question had a 5-point frequency response scale ranging from 0 (*never*) to 4 (*every day*), with a total score range of 0–64 and domain scores ranging from 0–16. A high score of CPQ_11-14_ indicates a lower OHRQoL. Severe impact on OHRQoL was defined as one or more items scoring 3 or 4 on the CPQ_11-14_.

DFA was measured using the validated Children’s Fear Survey Schedule-Dental Subscale (CFSS-DS) [24]. The 15 questions had response alternatives between 1 (*not afraid*) and 5 (*very afraid*), with total scores ranging from 15 to 75, where a high score indicates more DFA. Patients with a CFSS-DS score exceeding the cut-off value of 32 were classified as experiencing DFA [25]. When an item score was missing, the mean score was calculated based on the general score for that item.

All patients completed the questionnaires at the clinic. The patients were all instructed to answer the forms without any support from their caregivers.

#### Statistical methods

Statistical analyses were performed using IBM SPSS Statistics version 29.0 (Statistical Package for the Social Sciences; SPSS, Chicago, IL, USA). Chi-Square tests assessed categorical data. Independent T-tests and paired sample *T*-tests were used to compare mean scores, while Mann–Whitney U test was used to compare median scores. A *p*-value of less than 0.05 was considered statistically significant. The normality of the data was assessed using the Shapiro–Wilk test, with results guiding the choice of parametric or non-parametric statistical analyses.

A multiple linear regression analysis examined whether the sedation method affected OHRQoL and DFA at T1 while controlling for baseline values. CPQ_11-14_ and CFSS-DS scores at T1 were the dependent variables, with the sedation method as the independent variable, and baseline CPQ_11-14_ and CFSS-DS scores as covariates. Beta coefficients (β) and *p*-values were reported.

## Results

**Figure 1:**
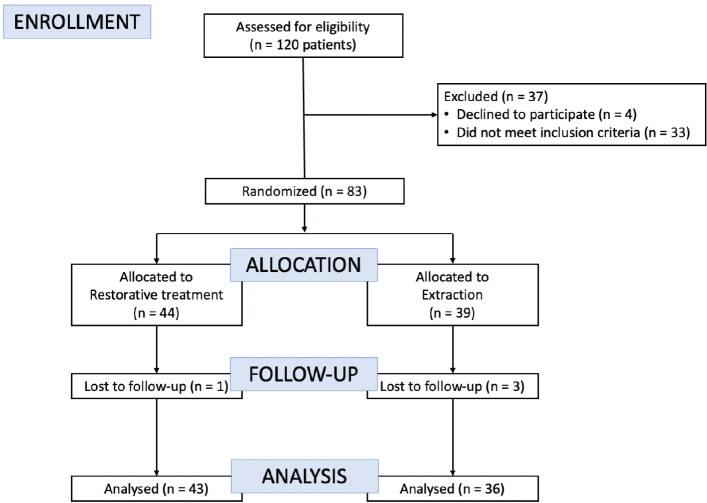
Flow-chart of patients invited to participate.

A total of 79 patients were included in the analysis, comprising 43 patients in the restorative treatment group and 36 patients in the extraction treatment group (Figure 1). There were no differences in gender distribution between the treatment groups. In addition, prior to treatment, there were no differences in the number of decayed, extracted, and filled teeth in the primary dentition (deft) between patients randomized to restorative treatment and those randomized to extraction treatment. (Table 2). Regarding sedation requirements among patients randomized to restorative treatment, 15 received treatment without sedation, 27 received sedation (benzodiazepine and/or nitrous oxide), and 1 was treated under GA. In the extraction treatment group, 2 were treated without sedation, 22 received sedation, and 12 were treated under GA. The Shapiro–Wilk test indicated that the difference between T0 and T1 of the CPQ_11-14_ score in the restorative treatment group, deviated significantly from normality (*p* = 0.006), whereas the other variables, including the CPQ_11-14_ score in the extraction treatment group and CFSS-DS score in both groups (*p* > 0.05), did not. Therefore, both parametric and non-parametric statistical analyses are used.

**Table 2:** Demographic and clinical characteristics of patients in the restorative treatment and extraction groups at baseline (T0).

	Restorative treatment	Extraction treatment	*p*
Number of participants	43	36	N/A
Sex; *n* (%)			
Female	22 (51)	23 (63)	0.363^1^
Male	21 (49)	13 (37)	
Number treated FPMs/patient; *n* (%)			
1	14 (32.6)	14 (38.9)	
2	17 (39.5)	12 (33.3)	
3	8 (18.6)	6 (16.7)	
4	4 (9.3)	4 (11.1)	
deft; mean (SD)	0.6 (1.5)	0.7 (1.8)	0.432^2^

1 Chi-Square tests,

2 Independent T-tests.Values in bold represent statistically significant association (*p* ≤ 0.05). *p*-value: probability value; *n*: number; FPM: first permanent molar; SD: standard deviation; deft: decayed, extracted, and filled primary teeth; N/A: not applicable.

### Oral health-related quality of life

At T0, the mean CPQ_11-14_ score for all the patients was 10.7 (SD 7.7). The order of domains from the highest to lowest score was as follows: Oral symptoms, Functional limitations, Emotional well-being, and Social well-being. No difference was observed between girls (mean 11.2, SD 7.4) and boys (mean 9.9, SD 8.1; *p* = 0.214).

At T1, the mean CPQ_11-14_ score for the patients was 8.6 (SD 6.7). Like T0, there was no difference between girls (mean 10.1, SD 7.1) and boys (mean 8.1, SD 6.9; *p* = 0.109). There was also no difference in the mean CPQ_11-14_ scores between patients who received restorative treatment and those who underwent extraction (Table 3).

**Table 3:** Mean (SD) and median (range) score of CPQ_11-14_ (range 0–64), each domain (range 0–16), and CFSS-DS (range 15–75) at T0 and T1 stratified between each treatment group.

		Restorative treatment (*n* = 37)		Extraction treatment (*n* = 36)		*p*-value
		Mean (SD)	Median (range)	Mean (SD)	Median (range)	(mean^1^ / median^2^)
Baseline (T0)	CPQ_11-14_: Total score	9.2 (7.3)	8.0 (32)	12.2 (7.7)	11.5 (30)	0.101 / 0.062
	Oral symptoms	3.6 (2.9)	3.0 (11)	4.9 (2.9)	4.0 (14)	0.119 / 0.095
	Functional limitations	2.4 (2.5)	2.0 (10)	3.8 (3.0)	3.5 (11)	**0.041** / **0.034**
	Emotional well-being	1.9 (2.4)	1.0 (10)	2.4 (2.6)	2.0 (11)	0.365 / 0.201
	Social well-being	1.1 (1.8)	0.0 (6)	1.1 (1.8)	0.0 (8)	0.862 / 0.722
	CFSS-DS	24.1 (8.5)	21.0 (39)	25.3 (7.4)	24.0 (32)	0.504 / 0.211
Follow-up (T1)	CPQ_11-14_: Total score	8.9 (7.3)	7.0 (32)	9.6 (6.7)	8.0 (32)	0.671 / 0.418
	Oral symptoms	3.3 (2.7)	3.0 (10)	4.6 (2.4)	4.0 (8)	**0.020** / **0.012**
	Functional limitations	2.1 (2.5)	1.0 (10)	2.4 (2.3)	2.0 (9)	0.520 / 0.323
	Emotional well-being	1.9 (2.7)	1.0 (12)	1.6 (2.3)	0.0 (7)	0.534 / 0.570
	Social well-being	1.7 (2.1)	1.0 (7)	1.0 (2.1)	0.0 (8)	0.158 / **0.014**
	CFSS-DS	21.5 (5.5)	21.0 (20)	23.1 (6.8)	21.5 (30)	0.268 / 0.326

*p*-value: probability value; SD: standard deviation.

1 T-test; ^2^Mann–Whitney U Test.Values in bold represent statistically significant association (*p* ≤0.05).

The mean CPQ_11-14_ score for all patients at T0 was 10.7 (SD 7.7), and at T1 it was 8.6 (SD 6.7), with a difference seen between T0 and T1 (*p* = 0.033). The ranking of domains remained unchanged between T0 and T1. Among patients who were randomized to restorative treatment, there was no difference in CPQ_11-14_ scores from T0 to T1 at group level (*p* = 0.137), or the individual level (*p* = 0.260). Conversely, for patients who underwent extraction, CPQ_11-14_ scores decreased at the individual level, with lower scores at T1 compared to T0 (*p* = 0.030), although this reduction was not statistically significant at the group level (*p* = 0.065) (Table 3). A multiple linear regression analysis showed that the sedation method did not influence the CPQ_11-14_ score at T1 while controlling for baseline values (β = 0.03; *p* = 0.981).

More patients in the extraction group (29%) reported being less affected at T1 than at T0, in comparison to patients in the restorative group (8%), (*p* = 0.027; Chi-square test).

### Dental fear and anxiety

At T0, the mean CFSS-DS score for the patients was 24.6 (SD 8.0). Prior to treatment, a difference in CFSS-DS scores was observed between the genders, with girls showing a higher mean score (26.1, SD 9.1) compared to boys (23.0, SD 6.0; *p* = 0.044). In contrast, there was no difference in DFA scores between patients allocated to the restorative treatment group and those allocated to the extraction group (Table 3). At baseline, patients treated without sedation had a mean CFSS-DS score of 21.8 (SD 5.2), those treated with sedation had a mean score of 24.8 (SD 8.3), and those treated under GA had a mean score of 28.2 (SD 9.3). Comparative analysis showed no difference in mean CFSS-DS scores between patients treated without sedation and those with sedation (*p* = 0.084) or between those treated with sedation and those treated under GA (*p* = 0.112). However, a difference was identified between the mean scores of patients treated without sedation and those treated under GA (*p* = 0.013).

At T1, the mean CFSS-DS score for the patients was 22.2 (SD 6.2). Girls continued to exhibit higher CFSS-DS scores (mean 24.6, SD 6.7) than boys (mean 19.1, SD 3.5; *p* < 0.001). There were no differences between patients randomized to restorative treatment compared to those randomized to extraction (Table 3). At T1, the mean CFSS-DS score for patients treated without sedation was 20.2 (SD 5.4), 22.1 (SD 5.3) for those treated with sedation, and 25.2 (SD 8.9) for those treated under GA. No differences were found between the mean scores of patients treated without sedation and those treated with sedation (*p* = 0.096), or between those treated with sedation and those treated under GA (*p* = 0.057). Nonetheless, a difference remained between the mean scores of patients treated without sedation and those treated under GA (*p* = 0.031). A multiple linear regression analysis showed that the sedation method did not influence the CFSS-DS score at T1 while controlling for baseline values (β = 1.18; *p* = 0.256). The reduction in CFSS-DS scores from T0 to T1 was seen in both patients randomized to restorative treatment (mean difference 2.6, SD 8.1; *p* = 0.021) and those randomized to extraction (mean difference 2.3, SD 5.3; *p* = 0.007; paired T-test). Table 4 provides a ranking of item scores for both T0 and T1.

**Table 4:** The study patients’ ranking of CFSS-DS items at T0 and T1.

CFSS-DS items	T0		T1	
	Rank	Mean (SD)	Rank	Mean (SD)
The dentist drilling	1	3.0 (1.4)	2	2.1 (1.1)
Injections	2	2.7 (1.3)	1	2.3 (1.4)
Choking	3	2.1 (1.3)	4	1.7 (0.9)
Having to go to the hospital	4	1.8 (1.0)	3	2.0 (1.1)
The sight of the dentist drilling	5	1.7 (1.0)	6	1.5 (0.9)
The noise of the dentist drilling	6	1.6 (0.9)	7	1.4 (0.6)
Having somebody putting instrument in the mouth	6	1.6 (0.9)	9	1.3 (0.5)
Doctors	8	1.5 (0.9)	7	1.4 (0.6)
Dentists	9	1.5 (0.8)	11	1.3 (0.6)
Having someone examine your mouth	10	1.4 (0.8)	11	1.6 (0.9)
Having a stranger touching you	11	1.3 (0.9)	5	1.6 (0.9)
People in white uniforms	12	1.2 (0.7)	15	1.1 (0.3)
Having somebody look at you	13	1.2 (0.5)	9	1.3 (0.5)
Having the nurse/dentist clean your teeth	13	1.2 (0.5)	13	1.1 (0.4)
Having to open your mouth	15	1.1 (0.4)	13	1.1 (0.4)

CFSS-DS: Children’s Fear Survey Schedule-Dental Subscale; SD: standard deviation.

The score of each question ranged 1 to 5.

There were no differences in the proportion of patients scoring above the cut-off value of 32 between those who received restorative treatment (14%) and those who received extraction (14%; *p* = 0.960; Chi-Square tests).

## Discussion

This study shows that patients who underwent either restorative treatment or extraction of their FPM affected by molar incisor hypomineralization (MIH), have comparable self-reported OHRQoL and DFA at 11 years of age. Notably, this is the first study to examine long-term OHRQoL and DFA in patients treated with either restorative or extraction approaches for MIH-affected FPMs. These findings offer helpful insights into patient needs and contribute to a better understanding of how to manage MIH in the clinical practice.

Concerning OHRQoL, no CPQ_11-14_ score differences were found at T1 between study patients who received restorative treatment, or patients who received extraction for their FPMs. Therefore, FPM loss due to MIH did not appear to lower OHRQoL in young patients in comparison to restorative treatment recipients. This statement cannot be verified, as no studies were found that analyze OHRQoL in young populations following the extraction of FPMs due to MIH or caries. Patients who underwent restorative treatment did not score a lower CPQ_11-14_ at T_1_ compared to study patients who underwent extractions. However, studies have shown that sealant treatment for FPMs with mild MIH decreases hypersensitivity and increases OHRQoL [26], while patients with severely affected MIH reported enhanced OHRQoL after restorations with glass hybrid material [27]. Currently, no MIH-specific instruments exist to measure OHRQoL, posing a challenge in capturing the unique experiences and impacts associated with the condition. The development of such a measure would allow for a more precise assessment of how MIH affects daily life, guiding both clinical decision-making and future research.

Girls in this study reported lower OHRQoL at the follow-up in comparison to boys, as confirmed by other studies examining PROMs related to MIH [27, 28]. This may be explained by gender differences in health perceptions and reporting, where girls generally report higher sensitivity to health issues and are more likely to express discomfort, pain, or emotional distress related to their oral health, whereas boys may underreport symptoms due to social norms [29]. Nonetheless, there are also studies showing no differences in scoring between the genders [5, 30].

Regarding DFA, study patients who received either extraction or restorative treatment exhibited similar proportions exceeding the DFA cut-off value. This suggests that despite extraction being more invasive, it does not significantly contribute to long-term DFA following treatment of MIH-affected FPMs. Notably, CFSS-DS does not have extraction as an item, which might be an intriguing area for further exploration. However, no other study has conducted a comparative analysis of DFA between patients who underwent restorative treatment and those who underwent extraction of FPMs with a poor prognosis. Furthermore, regression analyses showed that the type of sedation did not influence DFA or OHRQoL scores at follow-up when controlling for baseline values, indicating that sedation was not a confounding factor in patient-reported outcomes.

Like previous research, this study shows that girls experience higher DFA than boys [12 24 31]. Patients exhibit lower DFA as they get older, which aligns with other studies reporting that DFA tends to decline with age [13 14]. Despite this decline, items including injections and drilling are still ranked as the primary concern within both age groups. These findings support that gender and age may play a role in the development and perception of DFA, underscoring the importance of considering gender-specific factors when providing dental care and DFA management. However, it is essential to note that DFA is a complex issue and can be influenced by diverse factors such as past experiences, personality traits, and cultural beliefs. Overall, these results emphasize addressing DFA in all patients, regardless of the specific treatment planned, and the need for continued support and management to ensure patients receive necessary dental care without pain.

The sample size of 79 patients in this study limits the generalizability of the findings to a broader population. Although the initial power calculation indicated the need for 70 patients per group, the COVID-19 pandemic substantially hindered recruitment efforts. This study should be regarded as an exploratory effort aimed at identifying potential trends and informing future, more extensive research. Following the pandemic, further recruitment was not possible as it would have resulted in an excessive time range of patient recruitment, compromising the study’s internal validity. As a result, the study was underpowered to detect smaller effect sizes that might have been observable with a larger sample. Nevertheless, the participants were evenly distributed by gender and geographically across multiple regions, and the data obtained remains important for further analyses. This is the first trial comparing restorative treatment and extraction for FPMs with severe MIH. Although the follow-up period may restrict the ability to assess long-term treatment outcomes, further follow-ups are planned. Despite its short-term nature, this investigation provides valuable insights into the immediate impact of FPM treatment on children with MIH. Intra-rater agreement scores such as Cronbach’s alpha were not calculated, which represents a limitation in terms of assessing internal consistency in MIH diagnosis. Further research with larger sample sizes and longer follow-up periods is needed to validate these findings and explore additional factors influencing OHRQoL and DFA in this population.

## Conclusions

The findings of this study showed that extraction, compared to restorative treatment, does not negatively impact OHRQoL or increased DFA in patients with FPM affected by MIH.
